# Surgical treatments for women with stress urinary incontinence: a systematic review of economic evidence

**DOI:** 10.1186/s13643-020-01352-3

**Published:** 2020-04-20

**Authors:** Mehdi Javanbakht, Eoin Moloney, Miriam Brazzelli, Sheila Wallace, Muhammad Imran Omar, Ash Monga, Lucky Saraswat, Phil Mackie, Mari Imamura, Jemma Hudson, Michal Shimonovich, Graeme MacLennan, Luke Vale, Dawn Craig

**Affiliations:** 1 Institute of Health & Society, Newcastle University, Baddiley-Clark Building, Richardson Road, Newcastle upon Tyne, NE2 4AX UK; 2 Health Services Research Unit, University of Aberdeen, Aberdeen, UK; 3 Guidelines Office Methodology Supervisor, European Association of Urology, Arnhem, Netherlands; 4 University Hospitals Southampton Foundation Trust, Southampton, UK; 5 Aberdeen Royal Infirmary, Aberdeen, UK; 6 Scottish Public Health Network, NHS Health Scotland, Glasgow, UK

**Keywords:** Systematic review, Economic evaluation, Surgical treatments, Stress urinary incontinence

## Abstract

**Background:**

Surgical interventions for the treatment of stress urinary incontinence (SUI) in women are commonly employed following the failure of minimally invasive therapies. Due to the limited information available on the relative cost-effectiveness of available surgeries for treating SUI, a de novo economic analysis was conducted to assess costs and effects of all relevant surgeries. To inform the economic analysis, the objective of this review was to identify and assess the quality of existing economic evaluation studies on different surgical interventions for the treatment of SUI in women.

**Methods:**

The following databases were searched during the review process: Medical Literature Analysis and Retrieval System Online (MEDLINE), MEDLINE In-Process, Excerpta Medica Database (Embase), National Health Service Economic Evaluation Database (NHS EED), and Health Management Information Consortium and Cost-Effectiveness Analysis Registry (CEA registry). The key criteria for inclusion were that the study population included women with SUI and that the surgical interventions considered were utilised as either a primary or a follow-up surgery. The review included only full economic evaluations. Studies were quality assessed using the Drummond checklist for economic evaluations. No quantitative synthesis of the results by meta-analysis was conducted due to the high methodological heterogeneity.

**Results:**

Twenty-six economic evaluations were included, of which 13 were model-based analyses. Surgical treatments assessed most frequently were mid-urethral slings and open and laparoscopic colposuspension. There were some differences in the methodological approaches taken, including differences in type of economic analysis, perspective, time horizon, types of resource use, and costs and outcomes that were included in the analysis. The majority of studies conducted a cost-utility analysis from a health system perspective and applied a time horizon of between 1 and 5 years. The cost-effectiveness results suggest that single-incision mini-sling and mid-urethral slings are among the most cost-effective options.

**Conclusions:**

The review has shown that methods used for the economic evaluation of surgical treatments for SUI vary widely in terms of study design, analysis type, compared alternatives, time horizon, costing methodologies and effect outcomes. Future economic evaluation studies on surgical treatments for SUI may be improved by the application of available guidelines.

**Systematic review registration:**

Registered in PROSPERO in 2016, CRD42016049339

## Background

Stress urinary incontinence (SUI) is the involuntary leakage of urine due to any physical activity that puts pressure on the bladder, such as exercising, sneezing, coughing, laughing, or bending over [1]. SUI in women is a distressing condition, which can reduce their quality of life. Additionally, it can result in a large economic burden. The prevalence of SUI in women varies from 20 to 50% over a lifetime but is seen more often in women who have had children and in older women (above 40 years old) [2, 3]. Surgical treatment is usually recommended when conservative treatments have failed to control the condition [4]. Currently, there are various different types of surgical treatments for SUI including anterior vaginal repair or anterior colporrhaphy (anterior repair), bladder neck needle suspensions (bladder neck needle), open abdominal retropubic colposuspension (open-colpo), laparoscopic retropubic colposuspension (lap-colpo), traditional sub-urethral retropubic sling (trad-sling), retropubic mid-urethral sling (retro-MUS), transobturator mid-urethral sling (transob-MUS), single-incision sling procedures (single-incision sling) and peri-urethral injectable bulking agents (injectable agents). Each of these surgeries can be conducted using different techniques.

The various different types of surgical operations available, the different techniques used to perform these operations and the lack of a consensus among surgeons regarding which approach to use make it challenging to establish which procedure should be used to treat SUI. Although synthetic slings placed in a mid-urethral location are now often regarded as the standard of care [5], there is limited evidence that indicates that any one of the aforementioned procedures should definitively be used over the others based on safety, efficacy and cost-effectiveness. Economic analyses are an important basis for determining the cost-effectiveness of alternative treatments and interventions. In order to be considered useful for informing decision-making, there are certain criteria that all economic evaluation studies should fulfil. Any departure from these criteria means that results will not be generalizable and the strength of the study findings will be weakened [6]. A well-conducted economic evaluation should consider all interventions routinely used in the health system. The effect that an intervention has on all relevant costs should be considered. This includes not only the direct cost of the intervention, but also its effect on healthcare costs and all the expenditures incurred by patients. The costs relevant to the decision makers and study perspective should be considered when valuing costs. Where costs and benefits occur beyond a 1-year time horizon, they should be discounted to reflect the lower economic value of an expense that is delayed and the higher value of a benefit that is realized earlier. The time horizon should be of sufficient duration to capture all important differences in costs and outcomes between the interventions being compared. The clinical effectiveness estimates should be based on a systematic review and meta-analysis of results from randomised clinical trials (RCTs), or at least a single RCT, or, where this is not possible, appropriate robust evidence. Data to estimate health-related quality of life values should be reported by patients and/or carers involved in the individual studies.

There are currently several economic evaluation studies that have been conducted to evaluate the cost-effectiveness of different surgical treatments for SUI. However, it is unclear if newly available treatments such as retropubic mid-urethral sling, single-incision sling and injectable bulking agents really result in equivalent or better cost and health outcomes than older operations that were previously available (such as anterior vaginal repair or the different types of colposuspension). In order to enable both evidence-based choices about surgical effectiveness and to allow impartial counselling of women regarding the possible consequences of the alternative surgical operations for the management of SUI, it is essential to collect reliable evidence in a systematic and transparent manner. As part of a wider study exploring the effectiveness and cost-effectiveness of different surgical treatments for SUI in women [7], a systematic review of economic evidence was required. The aims of this review were to provide a summary of existing trial and model-based economic evaluation literature on currently available surgical interventions for the treatment of SUI/stress-predominant mixed urinary incontinence (MUI) (combination of SUI and urge urinary incontinence (UUI)) in women and to highlight key strengths and weaknesses of the identified studies in order to support future research.

## Methods

### Search strategy

The systematic review was conducted according to the general principles of the Centre for Reviews and Dissemination’s (CRD) guidance for undertaking reviews in health care [8], the recommendations of the Cochrane Handbook for Systematic Reviews [9] and the NICE guide to the methods of technology appraisal [6] and was reported according to the Preferred Reporting Items for Systematic Reviews and Meta-Analyses (PRISMA) [10] (registered in PROSPERO in 2016, CRD42016049339). The following databases were searched during the review process: Medical Literature Analysis and Retrieval System Online (MEDLINE), MEDLINE In-Process (from 1946 to January 2017), Excerpta Medica Database (Embase) (from 1974 to January 2017), National Health Service Economic Evaluation Database (NHS EED) (until 2015; the database hasn’t been updated since 2015), Health Management Information Consortium (from 1979 to January 2017) and Cost-Effectiveness Analysis Registry (CEA registry) (until January 2017). All databases were searched using the Ovid interface, except for the CEA registry which was searched through the CEA registry website. The search strategy used was tailored to each database (search terms are provided in the Additional file 1: Table S1 and Table S2). All searches were conducted in September 2016 and were updated in January 2017.

### Study eligibility

The key criteria for inclusion were that the study population included SUI (either patients with SUI only or stress-predominant MUI) and the surgical interventions considered were utilised as either a primary or follow-up surgery. The review included only full economic evaluations (trial- and model-based) as they provide information about costs and outcomes resulting from implementing each intervention, and hence represent the most relevant information for health care decision-making. A full economic evaluation was defined as a comparative study which included both costs and effects for two or more surgical interventions. No restrictions were placed on the publication timeframe or the study country, but only English language studies were included.

### Study selection

Two reviewers (MJ, EM), with experience in health economics, undertook the screening of titles and abstracts obtained through the search. All potentially relevant articles were obtained for full-text screening against the pre-defined selection criteria. Disagreements on the full-text articles were resolved through discussion between the two reviewers and, where necessary, by consulting a third author from the core team to make a final decision.

### Data extraction

A standard form was developed to extract the data from the included studies. The form was based on the Consolidated Health Economic Evaluation Reporting Standards (CHEERS) checklist [11]. Data extracted included study characteristics, country of study, target population, perspective of the economic evaluation, intervention and comparator(s) details, cost year and currency, study design (i.e. trial-based/model-based/other), analysis type (e.g. cost-effectiveness analysis (CEA), cost-utility analysis (CUA), cost-benefit analysis (CBA), cost-minimization analysis (CMA), cost-consequence analysis (CCA)), time horizon, model type and cycle length for model-based studies, discounting, resource use included, clinical effectiveness measure(s), quality of life measure(s), measure(s) of cost-effectiveness and type of sensitivity analysis conducted. Data extraction was undertaken by one reviewer, and all the extracted data were verified by the second reviewer.

### Quality assessment

As both trial- and model-based economic evaluation studies were included in the review, the quality of the economic evaluation studies was assessed using the Drummond checklist [12]. This is the standard checklist for reporting health economic evaluations, and it has been recommended in the guidelines developed for economic evaluation submissions to the BMJ. The quality assessment was done by two reviewers.

### Analysis

Meta-analysis was not conducted due to the high methodological heterogeneity. However, for all studies that reported total costs and quality-adjusted life-years (QALYs), we converted the total cost from reported values in the study (regardless of currency) to 2016 US$ using the EPPI-Centre Cost Converter [13]. The incremental net monetary benefit (INMB) for each of the surgical treatments was calculated as follows: incremental benefit × threshold − incremental cost. The threshold indicates the amount of money that each health system is prepared to pay for each extra QALY gained through different interventions. It was assumed that the threshold was US$ 50 K in the base-case, and US$ 30–40 K was assumed for sensitivity analyses. A positive INMB indicates that the intervention is cost-effective compared with the alterative at the given threshold. INMBs for each intervention compared with different interventions, as well as the range of estimated total costs of each intervention in different studies, are presented using forest plots.

## Results

### Literature search results

**Fig. 1:**
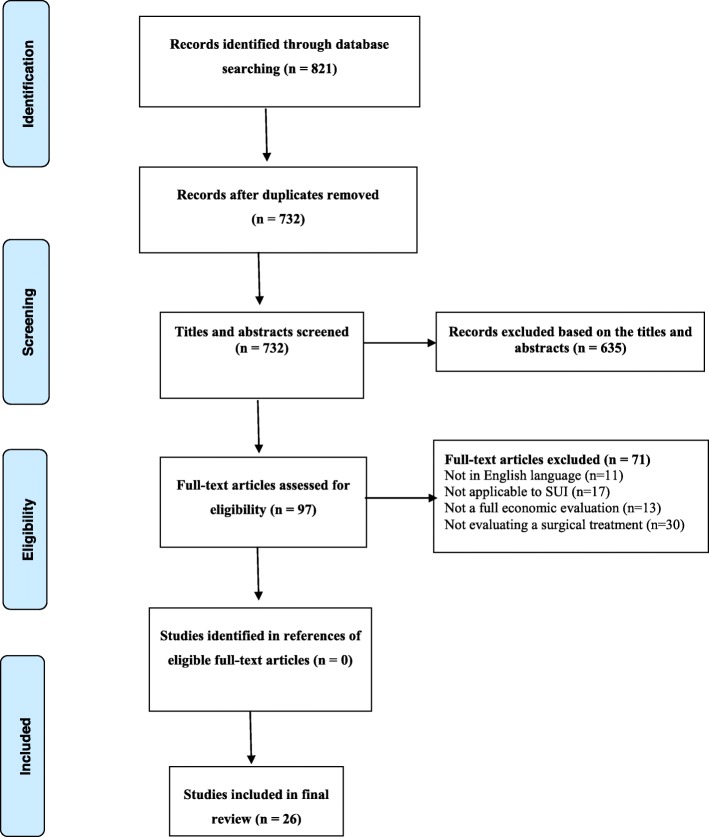
PRISMA Flow diagram showing study selection for the economic evaluations review

A total of 821 citations were identified from the original search, with 732 remaining after de-duplication. Following title and abstract screening, 97 studies remained. Full-text copies of these 97 studies were obtained for scrutiny against the full selection criteria and 71 were excluded as they did not meet at least one of the inclusion criteria. Reasons for exclusion included 11 were not in the English language, 17 were not applicable to SUI/stress-predominant MUI, 13 were not a full economic evaluation and 30 were not evaluating a surgical treatment. Therefore, 26 studies were included in the final review. All studies were conducted in women with SUI or MUI and included at least one of the surgical treatments for SUI/stress-predominant MUI. A flow diagram presenting the process of selecting studies can be found in Fig. 1. An overview of the key data extracted from these studies is presented in Table 1.

**Table 1:** Extracted data from the included studies

#	Name and year	Country	Study design	Analysis type	Intervention	Comparator/s	Perspective	Time horizon	Model type	CE measure	Intervention cost-effective?
1	Kunkle et al. (2015)	USA	Model based	CUA	Mid-urethral sling	Bulking agents	Healthcare system	1 year	Decision tree	Cost/QALY	No
2	Bargen et al. (2015)	USA	Model based	CUA	Mid-urethral sling	(1) Expectant management	Societal	Lifetime	Markov model	Cost/QALY	No
						(2) Pelvic floor muscle exercise					
						(3) Pelvic floor muscle exercise					
						(4) Vaginal cone/biofeedback					
						(5) Incontinence pessary					
3	Gupta et al. (2006)	UK	Model based	CUA	Duloxetine	(1) Pelvic floor muscle exercise	NR	2–5 years	Markov model	Cost/QALY	Yes
						(2) Surgery					
4	Jacklin et al. (2010)	UK	Model based	CUA	Tension-free vaginal tape	Duloxetine	Healthcare system	10 years	Markov model	Cost/QALY	Yes
5	Kilonzo et al. (2004)	UK	Model based	CUA	Tension-free vaginal tape	(1) Open colposuspension	Healthcare system	10 years	Markov model	Cost/QALY	Yes
						(2) Laparoscopic colposuspension					
						(3) Traditional sub-urethral sling procedures					
						(4) Peri-urethral injection therapy					
6	Laudano et al. (2013)	USA	Model based	CUA	Tension-free vaginal tape	Open Burch colposuspension	NR	10 years	Markov model	Cost/QALY	Yes
7	Oremus et al. (2003)	Canada	Model based	CEA	Collagen	(1) Retropubic suspension	Healthcare system	1 year	Decision tree	Cost/treated woman	No
						(2) Transvaginal suspension					
						(3) Sling procedure^a^					
8	Oremus et al. (2010)	Canada	Model based	CEA	Collagen	(1) Needle bladder neck suspension	Healthcare system	1 year	Decision tree	Cost/treated woman	Yes
						(2) Burch colposuspension					
						(3) Slings^a^					
9	Richardson et al. (2014)	US	Model based	CUA	Mid-urethral sling	(1) Continence pessary	Third-party payer	1 year	Decision tree	Cost/QALY	Yes
						(2) Pelvic floor muscle exercise					
10	Sand et al. (2014)	Canada	Model based	CCA	Transurethral radiofrequency micro modelling	(1) Tension-free vaginal tape	Healthcare system	3 years	Markov model	Cost	Yes
						(2) Trans-obturator tape					
						(3) Burch colposuspension					
						(4) Traditional bladder neck autologous sling					
11	Seklehner et al. (2014)	US	Model based	CUA	Retropubic- Mid-urethral sling	Transobturator- Mid-urethral sling	Healthcare system	10 years	Markov model	Cost/QALY	No
12	Weber et al. (2000)	US	Model based	CCA	Burch colposuspension	Sling procedure^a^	NR	10 years	Decision tree	Costs and clinical outcomes	N/A
13	Wu et al. (2007)	US	Model based	CUA	Burch colposuspension	Tension-free vaginal tape	Healthcare system	10 years	Markov model	Cost/QALY	No
14	Boyers et al. 2013	UK	RCT	CUA	Single-incision mini-sling	Tension-free vaginal obturator	NHS	1 year	NA	Cost/QALY	Yes
15	Castañeda et al. 2014	Spain	Retrospective data analysis	CMA	Single-incision mini-sling	Tension-free vaginal obturator	Healthcare system	1 year	NA	NA	Yes
16	Costantini et al. 2014	Italy	RCT	CUA	Mid-urethral sling	(1) Pelvic floor muscle exercise (2) Antimuscarinic treatment	National health service	3 months	NA	Cost/QALY	Yes
17	Dumville et al. 2006	UK	RCT	CUA	Laparoscopic colposuspension	Open colposuspension	NHS and PSS	6 and 24 months	NA	Cost/QALY	No
18	Hana et al. 2012	Bosnia and Hercegovina	Retrospective data analysis	CBA	Obturator tension-free vaginal tape	Vaginoplasty by Kelly	NR	6 months	NA	Cost/benefit	Yes
19	Lier et al. 2011	Canada	RCT	CUA	Trans-obturator tape	Tension-free vaginal tape	Public-payer	1 year	NA	Cost/QALY	Yes
20	Lier et al. 2016	Canada	RCT	CUA	Trans-obturator tape	Tension-free vaginal tape	Public-payer	5 years	NA	Cost/QALY Cost/% of patients with no SAE	Yes
21	Manca et al. 2003	UK and Ireland	RCT	CUA	Tension-free vaginal tape	Open Burch colposuspension	NHS	6 months	NA	Cost/QALY	Yes
22	Montesino-Semper et al. 2013	Spain	Prospective	CUA	Sub-urethral slings and prolapse meshes	No treatment	Public health care system	1 year	NA	Cost/QALY Cost /ICIQ-SF	Yes
23	Tiras et al. 2004	NR	Retrospective data analysis	CCA	Laparoscopic colposuspension extra peritoneal approach using mesh fixed with tacks	Laparoscopic colposuspension trans peritoneal approach using sutures	NR	25.7 months	NA	Cost	NA
24	VALPAS et al. 2006	Finland	RCT	CEA	Tension-free vaginal tape	Laparoscopic mesh colposuspension	Societal	1 year	NA	Cost/VAS Cost/UISS	Yes
25	Loveridge et al. 1997	Australia	Retrospective data analysis	CCA	Laparoscopic colposuspension	Open colposuspension	NR	NA	NA	NA	NR
26	Kung et al. 1996	Canada	Retrospective data analysis	CEA	Laparoscopic Burch colposuspension	Abdominal Burch colposuspension	Ministry of Health	1.2 - 2.7 years	NA	Cost/patient cured	NR

*CCA* cost consequences analysis, *CEA* cost-effectiveness analysis, *CUA* cost-utility analysis, *NA* not applicable, *NHS* National Health System; *NR* not reported, *PSS* personal social services, *QALY* quality-adjusted life years, *RCT* randomised control trial, *TOT* trans-obturator tape, *TVT* tension-free vaginal tape, UISS: Urinary incontinence severity score, *VAS* visual analogue scale

^a^Not specified the type of sling

### Basic characteristics of included studies

Seven studies were based in the US [14–20], six in the UK [21–26], six in Canada [27–32], two in Spain [33, 34], one in Australia [35], one in Bosnia and Herzegovina [36], one in Finland [37], one in Italy [38] and one in the Netherlands [39]. Thirteen studies were model-based analyses [14–23, 27–29], seven studies were within-trial evaluations [24–26, 30, 31, 37, 38], five were retrospective data analyses [32, 33, 35, 36, 40] and one was prospective non-randomised study [34]. Of the model-based studies, eight studies used a Markov model [14, 15, 17, 19, 21–23, 27] and five used a decision tree [16, 18, 20, 28, 29]. Sixteen studies were CUA [14–17, 19–26, 30, 31, 34, 38], four were CEA [28, 29, 32, 37], four were CCA [18, 27, 35, 40], one was a CMA [33] and one was a CBA [36]. Fifteen studies reported a health service perspective [17, 19–22, 24–29, 32–34, 38], two studies reported a societal perspective [14, 37], one study reported a third-party payer perspective [16], two studies reported a public payer perspective [30, 31] and six studies did not report a perspective [15, 18, 23, 35, 36, 40]. One study reported a lifetime time horizon [14], six studies reported a 10-year time horizon [15, 17–19, 21, 22], nine studies reported a 1-year time horizon [16, 20, 24, 28–30, 33, 34, 37], seven studies reported a 2–5 year time horizon [23, 25, 27, 31, 32, 38, 40] and one study did not report a time horizon [35]. Retro-MUS or transob-MUS were evaluated in 20 studies [14, 17, 19–24, 26–31, 33, 36–38, 41, 42] either as intervention or comparator, and 13 studies compared colposuspension procedures (either open-colpo or lap-colpo) with each other or other surgical treatments [18, 19, 22, 23, 25–27, 29, 32, 35, 37, 40, 41]. Four studies included injectable agents as either an intervention or a comparator [20, 22, 28, 29], and two studies have included single-incision sling [24, 33]. Other evaluated surgeries were trad-sling, bladder neck needle, and vaginoplasty by Kelly.

### Quality assessment

The completed Drummond checklist for the included studies is presented in Table 2. All of the included studies had a well-defined question posed in an answerable form and examined both the costs and effects of the alternative options [14–38, 40]. Only six of the included studies did not state a viewpoint for the analysis or place the study in any particular decision-making context [15, 18, 23, 35, 36, 40]. Three studies did not provide a comprehensive description of the competing alternatives, or only provided a comprehensive description of the intervention without focussing sufficiently on the comparator(s) [15, 17, 29]. Nineteen studies established the effectiveness of the surgeries through a randomised controlled clinical trial or systematic review of clinical evidence [14–19, 21–31, 37, 38]. Of the studies that did establish effectiveness, ten determined effectiveness through a randomised controlled clinical trial [16, 23–26, 29–31, 37, 38] and nine studies established effectiveness through an overview of clinical studies [14, 15, 17–19, 21, 22, 27, 28]. Seven studies used observational data or assumptions to establish effectiveness [20, 32–36, 40].

**Table 2:**
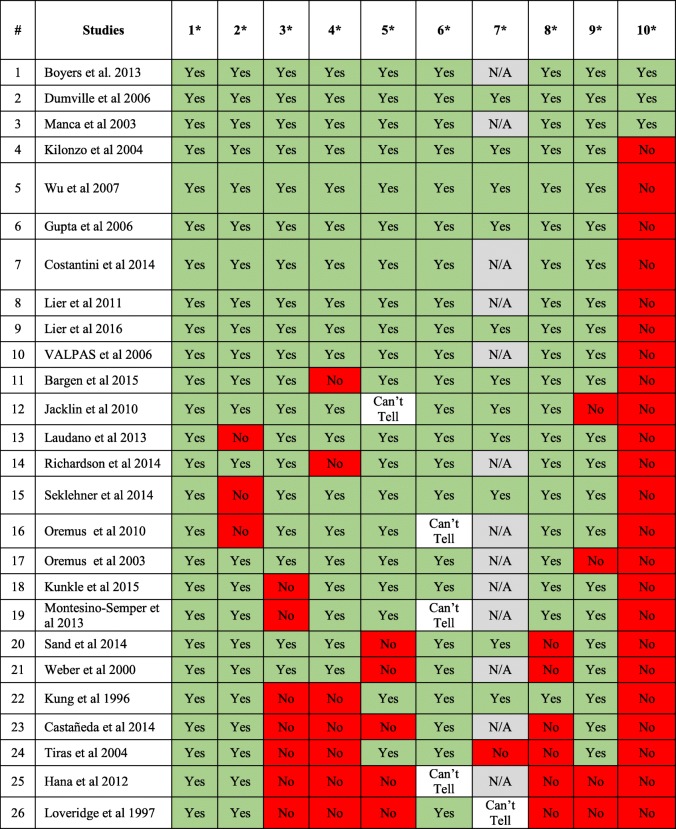
Methodological quality assessment of economic evaluations using Drummond’s checklist

*N/A* not applicable

*1.Was a well-defined question posed in answerable form? 2. Was a comprehensive description of the competing alternatives given? 3. Was the effectiveness of the programme or services established? 4. Were all the important and relevant costs and consequences for each alternative identified? 5. Were costs and consequences measured accurately in appropriate physical units? 6. Were the cost and consequences valued credibly? 7. Were costs and consequences adjusted for differential timing? 9. Was allowance made for uncertainty in the estimates of costs and consequences? 10. Did the presentation and discussion of study results include all issues of concern to users?

Most of the included studies identified the important and relevant costs and consequences of the alternatives being compared, except for seven studies [14, 16, 32, 33, 35, 36, 40]. Costs and consequences were covered from all relevant viewpoints (community or social viewpoint, and those of patients and third-party payers) in only three studies [14, 24, 37]. All of the included studies measured costs and consequences accurately in appropriate physical units, except for six studies [18, 27, 33, 35, 36, 40]. All of the included studies valued costs credibly and clearly identified the sources of all values, except for three studies where the sources of unit costs were not clearly identified [29, 34, 36].

**Fig. 2:**
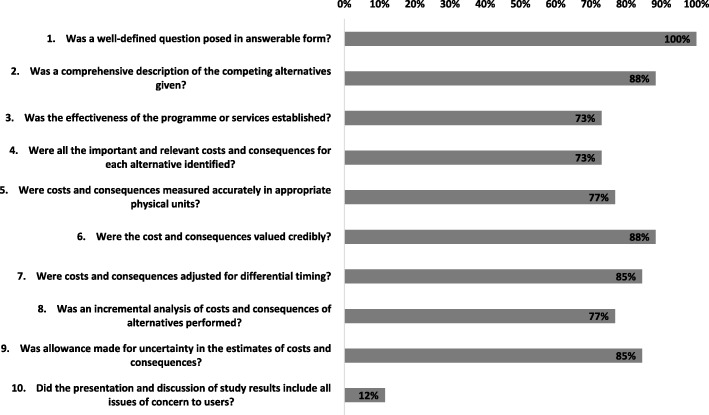
Percentage of “Yes” for each question of Drummond’s 10-point checklist for assessing economic evaluations

In eleven studies, costs and consequences were discounted appropriately [14, 15, 17, 19, 21–23, 25, 27, 31, 32]. In 13 studies, this was not applicable as the time horizon for the studies was ≤ 1 year. In one study, discounting was not reported [35]. Of the studies that applied discounting, only four did not provide any justification for the discount rate used [14, 22, 23, 27]. Six studies did not conduct an incremental analysis of the costs and consequences of alternatives [18, 27, 33, 35, 36, 40]. Only four studies did not make allowance for uncertainty in the estimates of costs and consequences [21, 28, 35, 36]. Only in three studies were all issues of concern to users and implementation discussed [24–26]. In all of the studies, other than five [18, 27, 33, 35, 40], the conclusions of the analysis were based on some overall index or ratio of costs to consequences. Sixteen studies discussed the generalisability of the results to other settings and patient/client groups [14, 16–19, 22, 24–29, 31, 34, 37]. Overall, included studies were of modest to high quality, and at least 73% of studies (*n* = 19) fulfilled nine out of ten criteria in the Drummond checklist [12]. Results of the quality assessment are provided in Fig. 2.

### Overall cost-effectiveness results

**Fig. 3:**
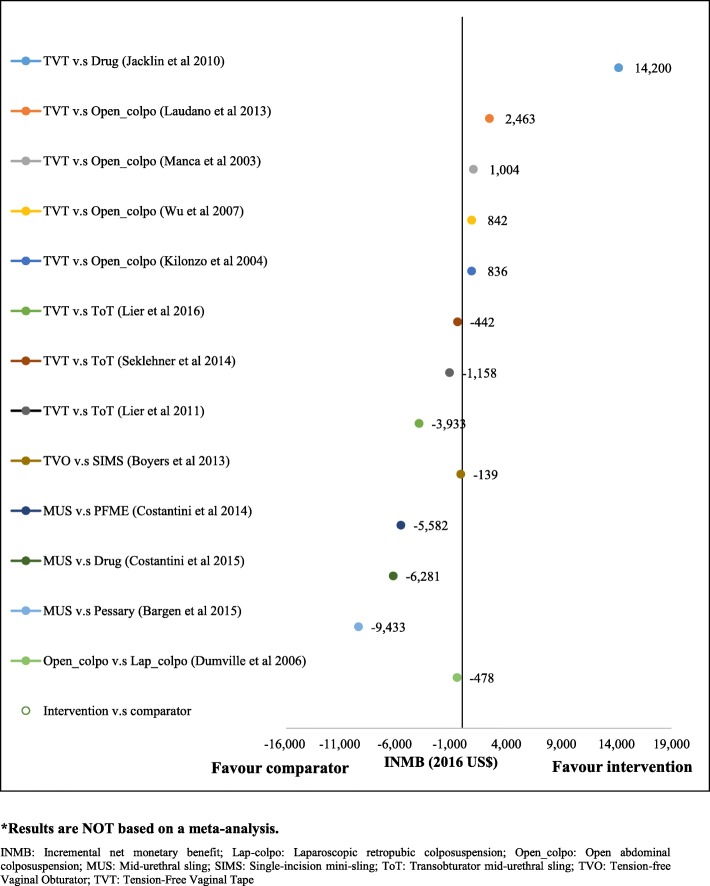
Incremental net monetary benefit for surgical interventions* (WTP = US$50 K). *Results are *not* based on a meta-analysis. *INMB* incremental net monetary benefit; *Lap-colpo* laparoscopic retropubic colposuspension; *Open_colpo* open abdominal colposuspension; *MUS* mid-urethral sling; *SIMS* single-incision mini-sling; *ToT* transobturator mid-urethral sling; *TVO* tension-free vaginal obturator; *TVT* tension-free vaginal tape

**Fig. 4:**
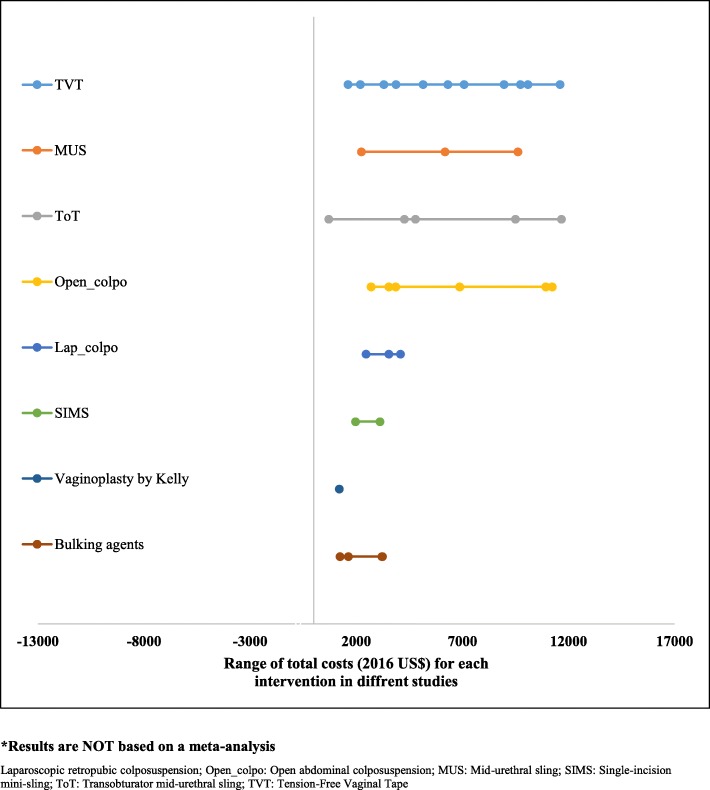
Estimated total costs* (2016 US$) for each intervention (circles indicate the number of papers/estimations). *Results are *not* based on a meta-analysis. *Lap-colpo* laparoscopic retropubic colposuspension; *Open_colpo* open abdominal colposuspension; *MUS* mid-urethral sling; *SIMS* single-incision mini-sling; *ToT* transobturator mid-urethral sling; *TVT* tension-free vaginal tape

Cost-effectiveness results are summarized in the last column in Table 1. Briefly, there are five studies that have compared tension-free vaginal tape (TVT) against open-colpo or lap-colpo and all of them concluded that TVT was more cost-effective than open-colpo [19, 22, 26, 37, 41]. All of these five studies were of high quality (Table 2). Two studies have compared TVT versus transobturator mid-urethral tape (TOT) [17, 30, 31]. The results from these two studies show that TOT is cost-effective compared with TVT. Two studies have compared single-incision sling against tension-free vaginal obturator and both of them concluded that single-incision sling is a cost-saving option compared to tension-free vaginal obturator [24, 33]. While the study by Boyers and colleagues [24] was of high quality, the study by Castaneda and colleagues [33] did not meet most of the Drummond checklist criteria, therefore was not a high quality economic evaluation. Lap-colpo was compared with open-colpo in three studies [25, 32, 35], and results show that lap-colpo is likely to be more cost-effective than open-colpo, especially over the long-term. Injectable agents have been compared with retro-MUS or transob-MUS in two studies [20, 28, 29], and results are contradictory as results from the study by Kunkle and colleagues [20] suggest that injectable agents are more cost-effective than MUS over a 1-year time horizon, while results from a study by Oremus and colleagues [29] show that surgery may be more cost-effective than collagen injections for the treatment of SUI. Overall, results of the economic evaluations suggest that single-incision sling and retro-MUS or transob-MUS are among the most cost-effective options followed by injectable agents and lap-colpo. Calculated INMB for different surgical treatments are presented in Fig. 3 and in Additional file 1: (Fig. S1 and Fig. S2). The range of estimated total costs for each intervention is also presented in Fig. 4.

## Discussion

To our knowledge, this is the first systematic review of the economic evidence on surgical treatments for SUI. We systematically reviewed and assessed the quality of 26 economic evaluations comparing nine different surgical treatments for SUI. The studies differed in terms of study design, analysis type, strategies compared, time horizon, costing methodologies and effectiveness outcomes. The surgical treatments assessed most frequently were retro-MUS or transob-MUS, and open-colpo and lap-colpo.

Although, as indicated in the results section, 73% of the included studies fulfilled nine out of ten criteria in the Drummond checklist [12], it could be argued that this checklist does not necessarily capture all components that are necessary for a methodologically robust economic evaluation. For instance, issues around time horizon of the analysis and the need to capture costs and outcomes for as long as those affected by the intervention are incurred are not considered in the Drummond checklist [12]. Similarly, the checklist does not explicitly consider perspective of the analysis among its criteria. In our own review, the time horizons of the identified studies were generally of insufficient length, with only one of the included studies assessing costs and consequences over a lifetime time horizon [14]. The majority of studies adopted ≤ 5 years’ time horizon which may not be of sufficient length to capture all the associated costs and effects of the surgical treatments. Only fifteen studies (58%) reported a standard outcome of incremental cost per QALY [14, 17, 19–26, 30, 31, 34, 38, 41, 42]. Furthermore, only two studies adopted the societal perspective. When the societal perspective is not used, it limits the generalizability of the cost-effectiveness findings. In several studies, not all relevant costs were included, which limits the applicability of the results.

Traditionally, in the area of health economic evaluation, a focus has been placed on ensuring that study assumptions and cost and health outcome measurement methodology have all been reported in a standardised manner [12]. Although some of the studies identified during this review included particular aspects of the guidelines [12] and provided useful research findings related to surgical treatments of SUI, only three studies (12%) were conducted entirely in accordance with the relevant guidelines [12] based on the criteria that were applicable. Based on results from this systematic review, it can be concluded that despite years of work on the development of guidelines for conducting and reporting economic evaluations in health care, work still needs to be done to ensure that future economic studies comparing surgical treatments for SUI adopt appropriate methodological approaches. This finding is consistent with the hypothesis put forward by Zwolsman et al. who claimed that economic evaluations in the area of stress urinary incontinence are often of low quality, with poor methodologies applied and inconsistent costing techniques [43].

Differences in methodologies applied, and the specific focus of most research questions, as well as differences in the reporting, design, assumptions, data included and perspective of the analyses, mean that it is difficult to say which of the alternative treatments is most cost-effective. Nonetheless, the results from the estimated INMB analysis suggest that single-incision sling and mid-urethral sling are among the most cost-effective options, followed by injectable agents and lap-colpo. However, a more robust conclusion on the cost-effectiveness of different surgical treatments can only be reached when the relative clinical effectiveness of all surgical treatments from available RCTs are assessed within a network meta-analysis and the results, along with other long-term data, are used within an integrated decision analysis model to estimate the comparative cost-effectiveness of all the surgical treatments. This work was conducted recently as part of the same UK study exploring the clinical and cost-effectiveness of nine different surgical treatments for stress urinary incontinence, which led to the systematic review presented here. In this study, a de novo economic analysis was conducted, and single-incision sling and retro-MUS were found to be the most cost-effective surgical interventions [7].

### Study limitations

As is the case with all systematic reviews, our study has limitations, which need to be considered. First, we included research published in English only and did not look at grey literature. Second, meta-analysis was not conducted due to the high heterogeneity among included studies. This makes it challenging to compare results of different economic evaluations and give an overall conclusion on the results. Instead, cost and effectiveness information for each intervention was extracted and/or calculated from those studies that had reported these values. Thirdly, any potential conflicts of interest related to funding sources associated with included studies were not considered. Finally, although a standard checklist (i.e. Drummond Checklist) was used to assess the methodological quality of the included studies, there are some issues that need to be highlighted. Firstly, it only examines the quality of included studies, and we were not able to judge the quality of reporting as this was beyond the scope of this review. Secondly, the same weights are given to all criteria in the checklist. One could argue that some items contribute more to potential bias of results than other items. However, it is difficult to find reliable sets of weight for each of the items in the checklist; therefore, this was not possible. Finally, as highlighted earlier, while the Drummond checklist is a perfectly acceptable tool for assessing the methodological quality of economic evaluations, it does not explicitly consider all issues of interest including time horizon and perspective of the analysis.

## Conclusions

This review identified the evidence base for economic evaluation of surgical treatments for SUI and assessed and highlighted the limitations and challenges of the included studies. This review has shown that there is wide variation in terms of study design, analysis type, compared alternatives, time horizon, costing methodologies and effect outcomes among the included studies. The quality of future health economic evaluation studies on surgical treatments for SUI may be enhanced by the rigorous application of quality guidelines, and the use of a societal perspective, common cost categories and appropriate measurement of health outcomes.

### Take home messages

- There are a number of different surgical interventions for the treatment of stress urinary incontinence in women.
- A systematic review was conducted to explore the evidence base of economic evaluations comparing surgical treatments for the condition and to assess their methodological quality.
- Twenty-six studies were included in the final review.
- Although 73% of the included studies fulfilled nine out of ten criteria on the quality assessment checklist used, there is scope for the methodological quality of future economic evaluations in this area to improve.

## Supplementary information

**Additional file 1 Table S1**. Search strategy. **Table S2**. The total number of studies retrieved by the individual databases is provided in table below. **Fig. S1.** Incremental net monetary benefit for surgical interventions (WTP=US$30K)—Results are NOT based on a meta-analysis. **Fig. S2.** Incremental net monetary benefit for surgical interventions (WTP=US$40K)—Results are NOT based on a meta-analysis

## Data Availability

There are no data to share; this article is based on a review of articles published in peer-reviewed journals.

## Abbreviations

SUI: Stress urinary incontinence
RCT: Randomised clinical trials
MUI: Mixed urinary incontinence
UUI: Urge urinary incontinence
CRD: Centre for Reviews and Dissemination
PRISMA: Preferred reporting items for systematic reviews and meta-analyses
CHEERS: Consolidated Health Economic Evaluation Reporting Standards
CEA: Cost-effectiveness analysis
CUA: Cost-utility analysis
CBA: Cost-benefit analysis
CMA: Cost-minimisation analysis
CCA: Cost-consequence analysis
QALYs: Quality-adjusted life-years
INMB: Incremental net monetary benefit
MUS: Mid-urethral sling
TVT: Tension-free vaginal tape
