# Supplementary material for: Surgical treatments for women with stress urinary incontinence: a systematic review of economic evidence
Source: Syst Rev. 2020 Apr 20;9:85. doi: 10.1186/s13643-020-01352-3 (PMC7169003; doi:10.1186/s13643-020-01352-3)
Supplement: Supplementary file 1 — Additional file 1 Table S1. Search strategy. Table S2. The total number of studies retrieved by the individual databases is provided in table below. Fig. S1. Incremental net monetary benefit for surgical interventions (WTP=US$30K)—Results are NOT based on a meta-analysis. Fig. S2. Incremental net monetary benefit for surgical interventions (WTP=US$40K)—Results are NOT based on a meta-analysis [file 13643_2020_1352_MOESM1_ESM.docx]

## Appendices

Table S1: SEARCH STRATEGY

| MEDLINE® and Medline in process | | | |
| --- | --- | --- | --- |
| No. | **Search term** | **Facet** | **Results** |
| 1 | exp models, economic/ | Economic evaluations | 12189 |
| 2 | *models, theoretical/ |  | 50817 |
| 3 | *models, organizational/ |  | 5483 |
| 4 | Markov chains/ |  | 11679 |
| 5 | monte carlo method/ |  | 23376 |
| 6 | exp decision theory/ |  | 10620 |
| 7 | (Markov* or monte carlo).ti,ab. |  | 49644 |
| 8 | econom* model*.ti,ab. |  | 2789 |
| 9 | (decision* adj2 (tree* or analy* or model*)).ti,ab. |  | 16267 |
| 10 | 1 or 2 or 3 or 4 or 5 or 6 or 7 or 8 or 9 |  | 145603 |
| 11 | URINARY INCONTINENCE/ | Disease | 19864 |
| 12 | URINARY INCONTINENCE, STRESS/ |  | 10063 |
| 13 | ((stress$ or mix$ or urg$ or urin$) adj3 incontinen$).tw. |  | 25719 |
| 14 | colporrhaphy.tw. |  | 519 |
| 15 | colpoperineoplast$.tw. |  | 28 |
| 16 | Sling procedure$.tw. |  | 898 |
| 17 | Sling$ procedure$.tw. |  | 905 |
| 18 | Bladder neck needle suspensions suspension$.tw. |  | 7 |
| 19 | Anterior vaginal repair$.tw. |  | 45 |
| 20 | 11 or 12 or 13 or 14 or 15 or 16 or 17 or 18 or 19 |  | 37510 |
| 21 | 10 and 20 | Final numbers | 139 |
| Embase | | | |
| No. | **Search term** | **Facet** | **Results** |
| 1 | URINE INCONTINENCE/ | Disease | 41326 |
| 2 | STRESS INCONTINENCE/ |  | 19497 |
| 3 | URGE INCONTINENCE/ |  | 6053 |
| 4 | MIXED INCONTINENCE/ |  | 1624 |
| 5 | ((stress$ or mix$ or urg$ or urin$) adj3 incontinen$).tw. |  | 39155 |
| 6 | URINARY URGENCY/ |  | 5368 |
| 7 | URINARY FREQUENCY/ |  | 6154 |
| 8 | ((urgency adj frequency) or (frequency adj urgency)).tw. |  | 1930 |
| 9 | ((urinary adj frequency) or (urinary adj urgency)).tw. |  | 3722 |
| 10 | colporrhaphy.tw. |  | 948 |
| 11 | colposuspension$.tw. |  | 1337 |
| 12 | Sling procedure$.tw. |  | 1554 |
| 13 | Sling$ procedure$.tw. |  | 1572 |
| 14 | Bladder neck needle suspensions suspension$.tw. |  | 7 |
| 15 | Anterior vaginal repair$.tw. |  | 83 |
| 16 | 1 or 2 or 3 or 4 or 5 or 6 or 7 or 8 or 9 or 10 or 11 or 12 or 13 or 14 or 15 |  | 69648 |
| 17 | statistical model/ | Economic evaluations | 149789 |
| 18 | exp economic aspect/ |  | 1386763 |
| 19 | 17 and 18 |  | 21900 |
| 20 | *theoretical model/ |  | 27446 |
| 21 | *nonbiological model/ |  | 4142 |
| 22 | stochastic model/ |  | 9838 |
| 23 | decision theory/ |  | 2696 |
| 24 | decision tree/ |  | 9013 |
| 25 | monte carlo method/ |  | 30151 |
| 26 | (Markov* or monte carlo).ti,ab. |  | 52002 |
| 27 | econom* model*.ti,ab. |  | 4018 |
| 28 | (decision* adj2 (tree* or analy* or model*)).ti,ab. |  | 22474 |
| 29 | 19 or 20 or 21 or 22 or 23 or 24 or 25 or 26 or 27 or 28 |  | 144098 |
| 30 | 16 and 29 | Final numbers | 269 |
| NHS Economic Evaluation | | | |
| No. | **Search term** | **Facet** | **Results** |
| 1 | URINARY INCONTINENCE/ | Disease | 31 |
| 2 | URINARY INCONTINENCE, STRESS/ |  | 34 |
| 3 | ((stress$ or mix$ or urg$ or urin$) adj3 incontinen$).tw. |  | 97 |
| 4 | colporrhaphy.tw. |  | 3 |
| 5 | colpoperineoplast$.tw. |  | 0 |
| 6 | sling procedure$.tw. |  | 4 |
| 7 | sling$ procedure$.tw. |  | 4 |
| 8 | Bladder neck needle suspensions suspension$.tw. |  | 0 |
| 9 | anterior vaginal repair$.tw. |  | 0 |
| 10 | 1 or 2 or 3 or 4 or 5 or 6 or 7 or 8 or 9 | Final numbers | 100 |
| Health Management Information Consortium | | | |
| # | **search terms** | **Facet** | **Results** |
| 1 | URINARY INCONTINENCE/ | Disease | 110 |
| 2 | URINARY INCONTINENCE, STRESS/ |  | 0 |
| 3 | ((stress$ or mix$ or urg$ or urin$) adj3 incontinen$).tw. |  | 174 |
| 4 | colporrhaphy.tw. |  | 0 |
| 5 | colpoperineoplast$.tw. |  | 0 |
| 6 | Sling procedure$.tw. |  | 0 |
| 7 | Sling$ procedure$.tw. |  | 0 |
| 8 | Bladder neck needle suspensions suspension$.tw. |  | 0 |
| 9 | Anterior vaginal repair$.tw. |  | 0 |
| 10 | 1 or 2 or 3 or 4 or 5 or 6 or 7 or 8 or 9 | Final numbers | 220 |
| Cost-effectiveness analysis registry | | | |
| # | **search terms** | **Facet** | **Results** |
| 1 | URINARY INCONTINENCE | Disease | 32 |
| 2 | STRESS URINARY INCONTINENCE |  | 14 |
| 3 | Mixed incontinence |  | 1 |
| 4 | URINARY |  | 72 |
| 5 | Incontinent |  | 3 |
| 6 | colporrhaphy |  | 0 |
| 7 | olposuspension |  | 6 |
| 8 | sling procedure |  | 1 |
| 9 | Bladder neck needle suspensions suspension |  | 0 |
| 10 | anterior vaginal repair |  | 0 |
| 11 | Sling |  | 14 |
| 12 | Incontinence |  | 50 |
| 13 | or/1-12 | Final numbers | 93 |

**Table S2: The total number of studies retrieved by the individual databases is provided in table below.**

| **Database** | **Numbers retrieved** |
| --- | --- |
| MEDLINE® and MEDLINE® In-Process | 139 |
| Embase^®^ | 269 |
| NHS Economic Evaluation Database | 100 |
| HMIC Health Management Information Consortium | 220 |
| Cost-effectiveness analysis registry | 93 |
| **Total** | **821** |

**Figure S1 Incremental net monetary benefit for surgical interventions (WTP=US$30K)- Results are NOT based on a meta-analysis. ***

***Results are NOT based on a meta-analysis**

INMB: Incremental net monetary benefit; Lap-colpo: Laparoscopic retropubic colposuspension; Open_colpo: Open abdominal colposuspension; MUS: Mid-urethral sling; SIMS: Single-incision mini-sling; ToT: Transobturator mid-urethral sling; TVO: Tension-free Vaginal Obturator; TVT: Tension-Free Vaginal Tape

**Figure S2 Incremental net monetary benefit for surgical interventions (WTP=US$40K)- Results are NOT based on a meta-analysis. ***

***Results are NOT based on a meta-analysis**

INMB: Incremental net monetary benefit; Lap-colpo: Laparoscopic retropubic colposuspension; Open_colpo: Open abdominal colposuspension; MUS: Mid-urethral sling; SIMS: Single-incision mini-sling; ToT: Transobturator mid-urethral sling; TVO: Tension-free Vaginal Obturator; TVT: Tension-Free Vaginal Tape
